# Pregnancy in an Asymptomatic Woman with Porencephalic and Arachnoid Cysts

**DOI:** 10.1155/2020/8885114

**Published:** 2020-11-18

**Authors:** Franco Pepe, Gianmarco Marchese, Gabriele Giuseppe Pepe, Elisabetta Zambrotta, Giulio Insalaco, Claudia Garraffo, Valeria La Rosa

**Affiliations:** ^1^Ospedale San Marco, Ostetricia e Ginecologia con Pronto Soccorso, Catania, Italy; ^2^Dipartimento di Neurochirurgia, UOV Careggi, Firenze, Italy; ^3^School of Anaesthesia and Intensive Care, University Hospital “G. Rodolico”, University of Catania, Catania, Italy

## Abstract

A 25-year-old woman presented to the obstetric clinic in her first pregnancy. The patient was accompanied by her mother who reported an episode of intracerebral hemorrhage after birth and also remembered access to the emergency department after generalized tonic-clonic seizure as an infant. She was not able to describe the therapy for seizure preventions and even when and why it was suspended, but she affirmed that no residual neurological consequences were detected in the following years. Actually, the pregnant woman was in good health without neurological symptoms nor assumed any therapy. A neurologist reviewed the patient's CT scan in which arachnoid cyst and porencephalic cyst were evident, and he assessed that no abnormalities were found in motor, sensory, and mental state examination. EEG did not show any epileptiform or seizure-like activity. No antiepileptic drug was prescribed due to the absence of symptoms since many years. The patient had no neurological symptoms during pregnancy or obstetric complications and delivered at term a healthy baby through a caesarean section. She breastfed, and after two years, the patient and the baby are healthy. The association of porencephalic and arachnoid cyst in pregnancy is an extremely rare neurological condition that needs a multidisciplinary counseling in pregnancy, but an uneventful course is possible.

## 1. Introduction

Porencephalic cyst, both congenital and acquired, is an accumulation of cephalorachidian fluid (CSF) in a cavity of the brain parenchyma probably from peri- or postnatal ischemic or hemorrhage vascular events or from traumatic brain injuries. It is reported in 3.5 per 100,000 live births [1]. Porencephalic cysts can be located in any lobe or lobes of the brain hemispheres and may be asymptomatic or present different symptoms: seizures, paresis, learning disability, mental retardation, and psychosis [2]. Moreover, clinical presentation is various as it is associated to differences in the sizes and sites of the lesions [3]: frontal lobe location may be associated with psychosis [4], whereas cerebral hemisphere involvement is associated with simple partial or generalized seizures [2]. Arachnoid cysts represent ~1% of all intracranial masses. They are benign and asymptomatic lesions occurring within the intracranial compartment (most common) as well as within the spinal canal. Only approximately 5% of arachnoid cysts cause symptoms due to mass effect. They are usually located within the subarachnoid space and contain CSF.

Arachnoid cysts can occur anywhere within the central nervous system, but they are most frequently (50-60%) located in the middle cranial fossa, where they invaginate into and widen the Sylvian fissure. In this location, they can be classified into three types based on their size with the Galassi classification. Of these, Galassi type I is the most common accounting for 78% followed by Galassi II and III with 19% and 3%, respectively [5]. Type I cysts are located in the anterior middle cranial fossa, small, and without symptoms; type II cysts stretch upward along the Sylvian fissure and can displace the temporal lobe; type III cysts are very large, occupying the entire middle fossa and can displace the temporal, parietal, and frontal lobes [6].

We describe a pregnancy in a woman with porencephalic and arachnoid cyst, an extremely rare association of neurological pathologies.

## 2. Case Report

A 25-year-old woman presented to the obstetric clinic during the 8^th^ week of her first pregnancy. Family history was negative for relevant diseases. The patient denied smoke, alcohol, or substance use, trauma, or surgical interventions. She was accompanied by her mother who described an episode of intracranial hemorrhage after birth with no subsequent cognitive or developmental sequelae. She also reported access to the emergency department after generalized tonic-clonic seizure as an infant, but she was not able to describe neither antiepileptic therapy nor reasons that led to its interruption. However, she did not report clinically significant neurological consequences.

A head CT scan performed in 2015 showed a very rare case of a left fronto-opercular porencephalic cyst associated with a large left temporobasal arachnoid cyst (Galassi II), communicating through the Sylvian fissure. In 2017, the patient underwent a follow-up MRI that confirmed no changes in the lesion previously described. The patient continued to be asymptomatic, and she decided to get pregnant.

At the time of presentation to our clinic, the woman was in good health. Physical exam was normal. Extensive laboratory routine was performed without any alteration, and mutations for genetic thrombophilia and autoimmunity were not detected.

**Figure 1:**
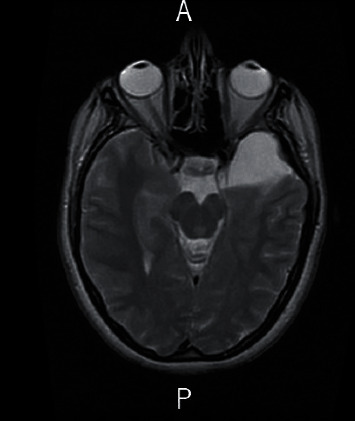
Axial T2-weighted view showing the big temporobasal arachnoid cyst communicating with the basal cisterns (Galassi II).

**Figure 2:**
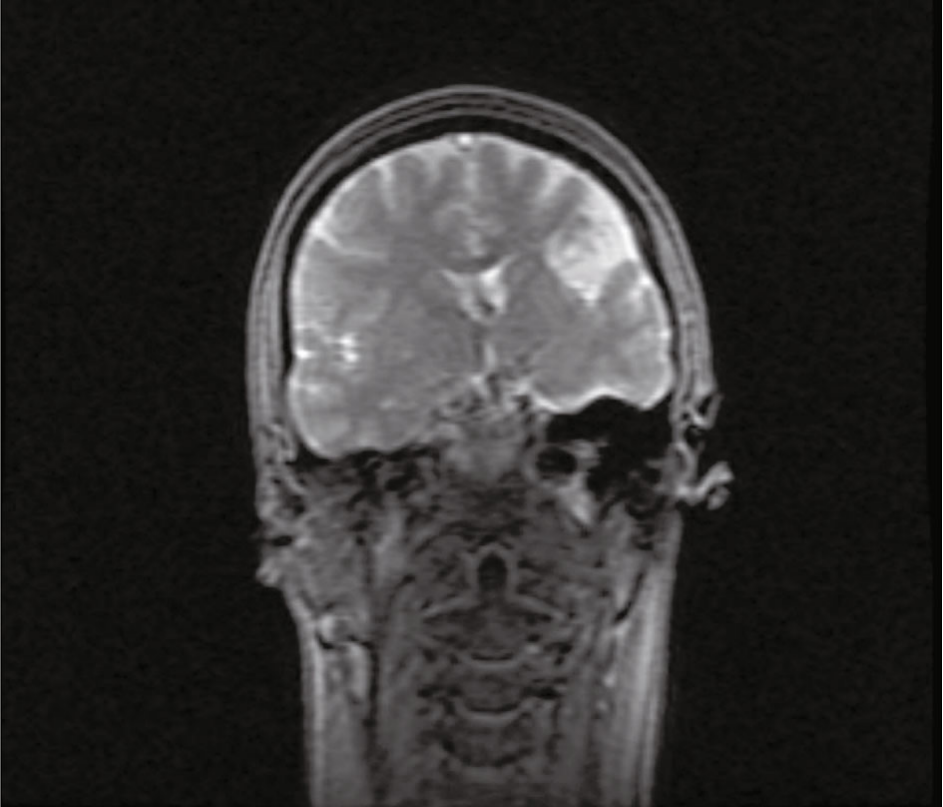
Coronal T2-weighted view showing the fronto-opercular porencephalic cyst with CSF inside.

**Figure 3:**
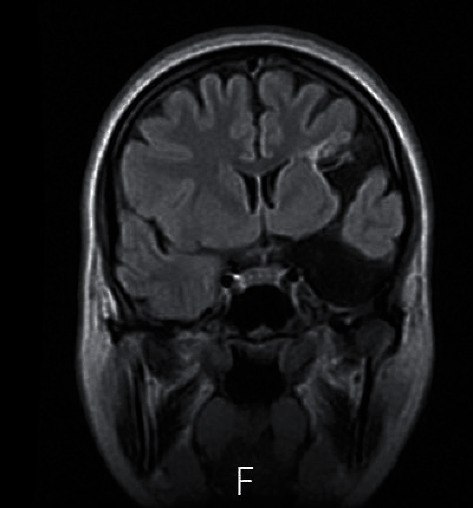
Coronal T1-weighted view showing the communication between the arachnoid cyst and the porencephalic cyst trough the Sylvian fissure, which makes this combination of conditions unique and characteristic.

The patient had no neurological symptoms nor assumed any therapy. A neurologist reviewed the patient's MRI scan in which porencephalic and arachnoid cysts were evident (Figures 1–3). The neurological examination was negative, and Mini Mental Test examination was normal. Electroencephalography did not show epileptiform or seizure-like activity. No antiepileptic drug was prescribed due to the absence of symptoms since many years. The neurosurgical consultation did not reveal any contraindications for the delivery through a caesarean section.

The medical team considered the woman and fetus at risk, she was monitored closely through pregnancy, and she was advised to report any relevant neurological symptoms. A peculiar aspect was counseling the patient for a possible recurrence risk of the porencephalic cyst and how to manage pregnancy, but family history was negative and no thrombophilic mutations were detected. The patient feared possible postpregnancy neurological complications, so she adopted a healthy lifestyle and avoided any stress or heavy physical activity.

The patient did not present any symptoms during pregnancy. The rarity of these lesions and the lack of experience in the literature, together with the clinical conditions of the patient, addressed our choice to a caesarean section delivery instead of a normal one, in order to avoid possible complications related to an increase of intracranial pressure (ICP). She delivered through caesarean section at the 40th week a healthy baby, as previously planned. The woman breastfed, and after two years, the patient and her baby are healthy.

## 3. Discussion and Conclusion

Porencephalic and arachnoid cysts are two distinct rare pathologies of the central nervous system that may be asymptomatic or have a wide array of clinical presentations. Patients with seizure are generally treated with antiepileptic drugs, and surgery is adopted only for refractory cases [7]. In our case, the two pathologies were associated and the patient reported no neurologic symptoms. No antiepileptic drugs were administered due to the long-term absence of symptoms.

Pregnancy was uneventful, but the lack of literature on this issue made the patient management very challenging. In our opinion, pregnancy should be considered at risk and carefully monitored for possible maternal neurological manifestations and for fetal well-being.

Anesthetic management may be challenging; although there is no clear contraindication to neuraxial anesthesia, a neurosurgeon should be consulted first. Epidural bolus analgesia may increase intracranial pressure (ICP), so it is advisable to administer continuous infusion [8].

Neuraxial anesthesia may be contraindicated only in case of evident intracranial pressure, as this may cause cerebral herniation, whereas it seems safe in an asymptomatic patient [9]. If general anesthesia is performed, adequate analgesia must be provided to blunt response to laryngoscopy that may increase ICP [10].

Arachnoid cyst is a sporadic pathology without significant recurrence risk. Also, most pathologies that cause porencephalic cysts (such as infections and monozygotic twinning) are sporadic, but a recurrence risk may exist if it is due to familial genetic thrombophilia or rare genetic mutation (for example, in COL4A1 gene) [11].

It may be advisable to have an international registry of patients with porencephalic and/or arachnoid cysts, because it may be useful to understand the needs, reproductive choice, and outcomes in these patients and to develop guidelines.
